# HIV Pre-exposure prophylaxis (PrEP) Modalities and Service Delivery Preferences Among Black Cisgender Emerging and Older Adult Women in Baltimore, Maryland

**DOI:** 10.21203/rs.3.rs-5112395/v1

**Published:** 2024-11-13

**Authors:** Deja Knight, Haneefa Saleem, Stefan Baral, Danielle German, Tiara C. Willie

**Affiliations:** Johns Hopkins Bloomberg School of Public Health; Johns Hopkins Bloomberg School of Public Health; Johns Hopkins Bloomberg School of Public Health; Johns Hopkins Bloomberg School of Public Health; Johns Hopkins Bloomberg School of Public Health

**Keywords:** PrEP, Cisgender Women, Black

## Abstract

**Background:**

Black cisgender women are disproportionately affected by HIV across the United States (US). Moreover, emerging adults continue to be significantly affected compared to women in older age groups. Yet in 2024, Black cisgender women and emerging adult women comprise a small fraction of HIV pre-exposure prophylaxis (PrEP) users in the US. This study examined PrEP modality, service delivery, and marketing and communication preferences by age among Black cisgender women in Baltimore, Maryland.

**Methods:**

Between October 2021 and April 2023, twelve Black cisgender PrEP-inexperienced emerging (18 to 29 years) and fourteen older (30 to 44 years) adult women were purposively recruited to participate in an in-depth interview. Interview topics included PrEP modality, service delivery, and marketing and communication preferences among the two currently approved modalities (oral and injectable) and the two modalities under investigation (ring and implant). Interviews were audio-recorded, transcribed verbatim, and analyzed using a combination of a deductive and inductive approach. Six follow-up member-checking interviews were also conducted.

**Results:**

Emerging adult women preferred oral PrEP, but older adult women preferred long-acting injectable (LAI) forms of PrEP. Oral PrEP was preferred because it was considered the most common modality for other medications, whereas LAI was preferred because it didn’t necessitate no daily administration. Emerging Black adult women reported challenges with adhering to the routine PrEP three-month follow-up period, such as transport, scheduling appointments, conflicts with school engagements, and being in a period of transition into adulthood where they experience structural changes (e.g., health insurance). Transport was the only reported challenge for older adult women for follow-up. Both age groups preferred longer follow-up periods to refill their PrEP prescriptions and to obtain PrEP from a trusted physician (e.g., OBGYN). Both groups of Black women expressed a preference for PrEP to be advertised through diverse means including social media campaigns, sexual health forums, peer groups on college campuses, and by featuring Black women in PrEP commercials.

**Conclusions:**

To improve PrEP equity and initiation among current and emerging PrEP modalities, it is crucial to better integrate the lived experiences and preferences of Black cisgender women and enhance their representation in PrEP messaging.

## Background

Black cisgender women comprise more than half of incident HIV cases among all women (1), yet, they make up a fraction of HIV pre-exposure prophylaxis (PrEP) users in the United States (US). Disparities in HIV infection for Black cisgender women persist throughout their life course, with the risk of acquiring HIV for Black cisgender women being 1 in 48 compared to a 1 in 880 risk for their white counterparts (2). HIV incidence and prevalence rates vary across age groups, with individuals aged 13 to 34 representing most (56%) incident HIV cases, including among women (3). New HIV diagnosis rates among women in different age groups are as follows: 13% for those aged 13 to 24, 27% for those aged 25 to 34, 26% for those aged 35 to 44, 19% for those aged 45 to 54, and 16% for those aged 55 and older (3). This variation in HIV incidence and prevalence across age groups highlights that U.S. Black women face different HIV risks and vulnerabilities at various stages of development. PrEP is an effective biomedical intervention that decreases the risk of HIV transmission through sex from a person living with HIV to a person living without HIV (4). PrEP is over 99% effective at reducing the sexual transmission of HIV when taken as prescribed (5). Since the majority of incident HIV infections are acquired through heterosexual contact for Black cisgender women, PrEP could help to reduce HIV infections among this group (6).

PrEP offers several methods for HIV prevention, with various forms approved and under study worldwide. Oral and long-acting injectable (LAI) forms of PrEP are the two modalities currently approved in the U.S., though emtricitabine-tenofovir is the only oral modality of PrEP currently approved for cisgender women. Oral PrEP is taken orally each day (7) and LAI has different formulations that are currently designed to be administered every eight weeks, though longer administration periods are under investigation (8). The vaginal ring, a silicone ring that is inserted into the vagina, has been studied and is approved in several African countries but remains under investigation in the US (9). The implant, which is inserted in the upper arm, is in early-stage (Phase I) clinical trials among human participants.

Prior research suggests Black cisgender women have diverse PrEP modality preferences that are influenced by a range of socio-structural factors. In prior studies, PrEP oral formulations, injectables, and implants were the most preferred modalities among Black women (10, 11). Oral formulations of PrEP were preferred for convenience and the injectable was preferred based on not taking a daily oral pill (11). The injectable has been reported as a preferred modality among Black women who are concerned about healthcare costs, PrEP-related stigma, or other structural factors that influence PrEP uptake (10). Other studies have demonstrated that all women preferred modalities that are easy to use, comfortable, longer lasting with reduced dosing schedules, and can be used discreetly (12, 13).

Black cisgender women may have specific PrEP service delivery and marketing preferences. Black women prefer to receive PrEP at diverse healthcare locations (e.g., OBGYN and primary care offices) (14), but also prefer to get PrEP information from their regular source of healthcare (15). Examining service delivery is important because a review of PrEP service delivery models among all women demonstrated that most models focus on high-risk groups, such as men who have sex with men, and may miss other populations, like Black women, who could benefit from PrEP (16).

Studies exploring PrEP modality, service delivery, and marketing and communication preferences in the U.S. by age are limited (17, 18), with none of the studies focusing on Black women. Age is important because Black emerging women have different HIV risk experiences compared to Black older adult women (19). Behaviors in adolescence for Black women can continue and impact behaviors and health outcomes in early adulthood (20). Building on prior research, this study used in-depth interviews to detail HIV PrEP modality, service delivery, and marketing preferences among HIV PrEP-naïve Black cisgender women still of reproductive age, focusing on emerging adult (EA) aged 18 to 29 and older adult (OA) women aged 30 to 44 women in Baltimore, Maryland, an *Ending the HIV Epidemic priority* area (21). For this manuscript, we define service delivery as “consisting of four key components: the population targeted for PrEP, the infrastructural setting of PrEP provision, PrEP providers, and the applied delivery channels to make PrEP available (16).” We also conceptualize marketing and communication as any PrEP advertisement materials, which is important to examine as PrEP messaging can influence Black women’s interest in PrEP (22–24).

## Methods

### Study Setting

We conducted this study in Baltimore, Maryland, a predominately Black or African American urban city, with Black non-Hispanic residents comprising 62.3% of the population (25). In 2019, Baltimore had an HIV incidence rate of 199 cases per 100,000 people, nearly 20 times the rate of the average diagnosis rate in Maryland (26). As a result of these disparities, Baltimore is a priority jurisdiction and Black women are a priority group in the National Ending the HIV Epidemic Plan (21). Baltimore, Maryland is unique in that women comprised 35.2% and Black people comprised 81.4% of HIV incidence cases, yet women only represent 13.2% of PrEP users in the city (26). This highlights the importance of intervening to prevent HIV for Black women, in Baltimore, Maryland, specifically.

### Participants

From October 2021 to April 2023, we interviewed Black cisgender PrEP-naïve women aged 18 to 44 who filled the following eligibility criteria: (1) self-identifying as African American or Black cisgender female; (2) self-reporting HIV-negative status with no previous HIV PrEP use; (3) currently residing within the city limits of Baltimore; and (5) sexual risk indicators for heterosexually active individuals such as having vaginal or anal sex with a male partner and inconsistent condom usage within the past six months (27).

### Procedures

We purposively sampled Black cisgender women based on age group: EA (n = 12) and OA (n = 14) Black women. To recruit, we placed flyers around Baltimore, on social media, and handed flyers out at in-person events where Black women were likely to attend. If eligible, Black women completed a one-time, in-depth, individual virtual interview that lasted between 45 to 60 minutes. Interview topics included PrEP modality, including the two currently approved modalities (oral formulations of PrEP and injectable) and the modalities currently being explored (vaginal ring and implant), PrEP service delivery, and PrEP marketing and communication preferences. Two Black cisgender women (the first author and a research assistant) completed all interviews, with the main interviewer also being from Baltimore. We gave participants a 45 USD Visa gift card for their participation. Interviews were audio-recorded and transcribed. All study materials were approved by [masked for review].

### Data analysis

We developed a codebook based on preference domains (i.e., PrEP modality, PrEP service delivery, and PrEP advertisement) and PrEP modalities (i.e., oral formulations of PrEP, injectable, ring, and implant). Sub-codes were inductively derived from a line-by-line reading of two transcripts by three research team members. Two members of the research team coded each of the remaining transcripts using the codebook. Bi-weekly meetings with the three Black cisgender women who were part of the coding team were used to discuss coding consistency and discrepancies, which were resolved through group consensus. After coding was complete, codes were analyzed by age group and were summarized into themes. Finally, emergent themes were compared between the two age groups.

Member-checking interviews were completed with six Black women from our initial sample. Each participant was invited to participate in a member-checking interview if they indicated that they could be reached for future research. The first three Black women in each age group was scheduled for and completed an interview. During the member-checking interviews, Black women were given a summary of findings within each theme and were asked if they findings sounds correct to them. They were asked to expand if the findings did not sound correct and were asked if anything was missing from the summary of findings. Additionally, Black women were asked about a typical doctor’s appointment and whether they discussed PrEP with their provider since the initial interview. Member-checking interviews lasted approximately 45–60 minutes. MAXQDA 2022 was used to organize and code the data (28).

#### Rigor in Qualitative Research

We utilized several strategies to ensure rigor and to improve quality: 1) prolonged engagement, 2) “rich” data and thick descriptions, 3) peer debriefing, and 4) member checking (29–31). We not only sought diversity among Black women concerning age, education, and income but also among lived experiences. Second, verbatim transcription of audio files and memos allowed for rich and thick descriptions of the data through the use of direct quotes within the resulting manuscripts. Third, we completed peer debriefing amongst the coding team during biweekly meetings where we discussed code applications, coded segments, and any disagreement among code applications. Rigorous documentation aided us in ensuring that we have dependability in our findings. Finally, we completed member-checking interviews to check the validity of the findings from the interviews. This process not only allowed participants to ensure that our interpretation of the data was correct but also allowed participants to expand on previous discussions.

#### Positionality Statement

The first author is a Black cisgender woman from Baltimore. Being from Baltimore, she was able to identify key places to recruit other Black cisgender women to participate in this study. She was also able to build rapport with the Black women who participated in this study. The two research assistants who helped to interview and code the data are also Black women.

## Results

Our sample included 12 EA Black women with an average age of 22 (range = 18 to 29) and 14 OA Black women with an average age of 36 (range = 30 to 42). Nine Black women were college students, seven aged 18 to 29 and two aged 30 to 44. EA Black women preferred oral PrEP, OA Black women favored the injectable, and both groups preferred longer follow-up periods and diverse marketing for PrEP. Below we present a more in-depth discussion of preferences reported by participants by modality,service delivery, and marketing and communication.

### Preferred PrEP Modality

Most EA Black women preferred oral formulations of PrEP with no one expressing a preference for the vaginal ring. Most OA Black women preferred the injectable. PrEP modality preferences aligned with Black women’s birth control preferences, as those women who had good experiences with a birth control modality expressed positive perceptions towards that same PrEP modality.

### Oral Formulations of PrEP

Oral formulations of PrEP were the preferred modality for most EA Black women. Some EA Black women felt the pill was “traditional” since many medications come in pill form and compared the pill to taking a daily vitamin, which was appealing.

I would say my preferred is the pill. I like the idea of taking a daily vitamin…it’s easier for me I guess you can say. Like the idea of it seems better than an injection or something that is pushing out the dosages or even a ring going inside of me... – Tanya, 19-year-old EA Black woman

However, some EA and many OA Black women did not like the idea of taking oral formulations. One major concern with taking oral formulations of PrEP for EA and OA Black women was the possibility of missing a dose. EA Black women with a daily routine (e.g., college students) preferred oral formulations of PrEP because they could easily incorporate it into their schedule, reducing the likelihood of forgetting to take it. However, some EA Black women noted that others might forget to take their pill because they may not perceive PrEP as essential in the same manner as medication used to treat a health condition.

I like that [PrEP pill] because I also take other medications that are daily pills, so that’s fine. But some people might forget to take it especially if it’s not something that they necessarily need…some people need to take medications to survive but being as though that PrEP is a preventative medication, [it is] not something that you necessarily need, people might be like, “Oh, I don’t need to take it today,” and I feel like that might reduce the effectiveness of it. – Jessica, 18-year-old EA Black woman

A few EA and OA Black women who reported taking daily medications to treat health conditions expressed a “negative relationship” with medications in the pill form.

I have a very negative relationship with pills, just because I have to take some for my blood thing. So, I do not like taking pills at all. I can do it. But I hate taking pills with a passion…having to do it every day. And two, like, because I have to take so many, it would just feel like an extra…I take a vitamin every day to keep my levels balanced with an allergy pill and then a muscle relaxer. So that’s already three pills every day…two to three every day because it depends on which medicine that I have to take... – Tamia, 21-year-old EA Black woman

### LAI Forms of PrEP

Most OA Black women reported a stronger preference for injectables but had similar concerns to those of EA women. Both groups of women thought this modality would be good for individuals who may forget to take pills. Similar to oral formulations of PrEP, EA Black women also felt injectables are also a “traditional” modality. The major benefit of the injectables for EA and OA Black women was that it would help people who do not like taking oral medications or who have challenges remembering to take daily medication.

I feel like if I could, I would just go ahead and take the shot because I know I’m not a frequent pill-taker. I mean I only take medication when I’m sick and to keep my allergies down, but I can’t even get myself to take that every day every season so if I was in a position where I would take the shot just to get it over with so I’m good for the month… – Shanice, 23-year-old EA Black woman

EA Black women stated that taking PrEP as an injectable was also said to benefit the privacy of Black women.

…an injectable is a lot more discreet than taking the oral pills. If you’re somebody that doesn’t want to have a bunch of your pills in your home let people know what you’re taking. An injectable, it would keep giving you your privacy. – Aniya, 29-year-old EA Black woman

Notably, when discussing each PrEP modality, many EA Black women said they do not prefer the injectables. Some EA Black women did not prefer injectables due to needle aversion, concerns about insertion procedures, and side effects. However, a few EA and OA Black women said they would take the injectable despite perceived challenges due to the benefit of HIV prevention.

I don’t like needles, but I feel like I could deal with it if it’s that much of a preventative measure… I’m not very scared of needles. So, if I have to get it, I’ll get it. It’s not a deal breaker for me… – Samantha, 22-year-old EA Black woman

Though side effects also remained a concern, the injectable gave two OA Black women confidence that PrEP would be in their system, especially since they did not have to actively thinking about taking a daily medication.

The benefit of the shot is you don’t have to take medicine every single day. There may be a fee, but at least you know it’s in your system, and you don’t have to be on your stuff about forgetting to take it. So, I feel like the shot would be the best modality to just get it over with. You get your shot, and you make the appointment and get it every month because you’d be less responsible to take it every day, but you’d be most responsible in knowing you’re tolerant to do it every day versus one time a month. – Shanice, 23-year-old EA Black woman

### Vaginal Ring

None of the EA Black women preferred the ring. In contrast, one OA Black woman preferred the ring over other modalities, although she also expressed concerns similar concerns to EA Black women. Some EA Black women said a benefit of the ring is being able to insert it and forget about it. However, this was not universally perceived as a benefit, as the thought of inserting a ring was described as a potential challenge by many EA Black women.

I would get the shot or take the pill before that [ring]. I’m not too fond of sticking things up my vagina like that, but I do tampons, and it took me a while before I did sometimes because I had to find the certain one that I like, and I like the pearl ones and for certain times, you know. So, I wouldn’t do the insert the vagina silicon thing… – Shanice, 23-year-old EA Black woman

Other concerns about the ring included questions of comfort and stability, how it would impact the use of menstrual products (e.g., menstrual cup), and how the ring works. There were also questions about how it protects you from HIV, why it only protects against HIV, and its impact on sexual intercourse. For OA Black women who had the ring form of birth control before, they did not like it during sex and questioned its efficacy.

My question is, if I’m going to stick something in me to protect against HIV, why am I not being protected against any other STI? Why just HIV? How did they protect the man? I know this is about females so that probably is not even a valid question, but that’s one of my next questions. But does it protect me if I’m a female having sex with another female? Still don’t like the vaginal one [ring]…I remember my times with the ring birth control modality—it raised too many questions. And I’m like, “I’m not trying to have a sex education right before we have sex.” So, I can just imagine that being an issue with the vaginal ring. – Tameka, 40-year-old OA Black woman

One EA Black woman said that she would not get the ring because she has post-traumatic stress disorder from a past medical experience.

I also would not do that one [ring]. I have PTSD from inserting things myself vaginally. I used to get a procedure where they would stick a catheter up your urethra…so I had to do a lot of surgeries to fix that when I was younger and stuff. So, anything that is vaginally inserted, I tend to [steer away]. – Angelica, 22-year-old EA Black woman

### Implant

EA and OA Black women shared perceived benefits of the implant such as its long-acting properties. Concerns of the implant mainly focused on implantation, migration, and removal. EA Black women’s concerns stemmed from experiences with birth control implants, while OA Black women’s concerns stemmed from experiences or stories with the intrauterine device birth control modality.

EA Black women expressed optimism about the implant but shared concerns that would impede them from using this modality. Two EA Black women stated they were scared of the implants because of a foreign body being inserted into the body and the possibility of the implant migrating into the body.

I wouldn’t do the implant simply because I do not want anything under my skin [hormonal contraceptive implant] …The [hormonal contraceptive implant] … move…every story that I’ve heard about somebody having it, it moved a little bit in my arm, and I can’t find it. Or one of my friends from school got the implant in her arm and they just lost it…And I’m like, I’m not doing that…if I get something, it’s something that I can choose to stop taking at whatever time I want to stop taking it. And not have to go make an appointment for somebody else to get it out. I don’t want anything in my body that’s not a liquid. – Samantha, 22-year-old EA Black woman

EA and OA Black women discussed the benefit of PrEP consistently being in you to disperse medication. OA Black women also discussed the benefits and drawbacks of implants. OA Black women liked that you do not have to concern yourself with daily medications, despite their concerns that something could go wrong with the implant based on experiences with the intra-uterine device (IUD) birth control, which was a common concern among Black women in this study.

I probably would take it simply because I’m familiar with the IUD, so I understand just having it in place, and, honestly, with this, I find it to be more of a safe modality. I still go talk to doctors and get checkups, so I know as long as I make sure whatever this is in place, and it’s working properly, and it was manufactured properly that things of that nature there’s a slim chance of things going wrong…but I know it’s there. – Stephanie, 30-year-old OA Black woman

Similarly, another OA Black woman reported mixed feelings about the implant but was willing to consider it based on a mix of positive and negative reviews from individuals who used intrauterine device as a birth control modality.

### Service Delivery Preferences

For service delivery, we explored potential challenges with follow-up appointments and prescription needs for PrEP. Both age groups cited transport as the main concern, with EA Black women also anticipating schedule and responsibility conflicts, and OA Black women anticipating mobility and childcare barriers. Both EA and OA Black women thought needing a PrEP prescription was normal, though EA Black women anticipated barriers to securing a PrEP prescription such as attending appointments with their mothers.

### Follow Up Burden

Most EA Black women said that they would be fine with going back to the doctor’s office every three months for follow-up.

…judging from how PrEP works, it’s like you said it’s preventing you from being exposed to HIV, one should be willing to be willing to spare some time to get it. So, I could sacrifice a day for it, for the whole scheduling and everything. – Cristina, 26-year-old EA Black woman

However, transport was a major concern for some EA and OA Black women. Women without cars often depended on public transport, which is unreliable and costly, and poses a perceived barrier for adhering to follow-up. Therefore, general convenience and proximity of the clinic was preferred for easier PrEP follow up.

I would not mind it [follow-up period], long as the clinic wasn’t far…I feel like long as the location is [convenient], then I don’t think that’s a bad thing…the busses only to the facility is a struggle…it’s the summer now, so it’s really hot and when you don’t want to deal with that, especially when you’re already depending on the appointment…it feels like you kind of dread the commute. So, if they could accommodate or sponsor rides there or something like that…wintertime it’s freezing, [it] would be nice [to have a ride] …that’d be one less thing to worry about... – Ariel, 32-year-old OA Black woman

For EA Black women with transport, scheduling was a challenge for follow-up. In particular, EA Black women in college discussed scheduling conflicts with school engagements and classes that may impede them from adhering to follow-up.

Testing every three months is kind of frequent for me. Yes, 9 times out 10, I would probably miss a couple of those appointments and have to reschedule, simply because I am always late to everything, and things pop up in my schedule kind of often. I have a car, but scheduling is crazy. I live with my mom, so I normally have to take her to places that she needs to go, I’m in school, and I work a full-time job. So, like, things just pop up out of nowhere that I have to take care of, and cancel at the last minute, and a lot of stuff like that. – Samantha, 22-year-old EA Black woman

However, OA Black women stated they would be fine with the follow-up process. They emphasized the importance of regular HIV testing to ensure overall health and well-being.

I would be okay with that [follow-up period]. Because one you might not know what the side effects are or how the medication is affecting your body, so you don’t know if it’s affecting your kidney function or if it is making stuff go haywire. So, I feel like you need to be checked, especially, when it’s such a new medication. – Latoya, 33-year-old OA Black woman

Additional follow-up considerations for OA Black women included physical mobility and ensuring minimal interference with work or childcare responsibilities.

### PrEP Prescription

EA and OA Black women thought needing a prescription for PrEP was normal, just like needing a prescription for birth control. They also expressed trust in their doctor to decide whether they are good candidates for PrEP.

I feel like that’s just a good step because our doctor handles all of our medicals because they can tell us how your body would react to the stuff that’s in PrEP…Like, I feel like I could talk to my doctor. I really trust my doctor to make decisions for my body because she has all of my medical records. So, like, let’s say something in PrEP that I’m allergic to, my body might react differently to it. – Tisha, 20-year-old EA Black woman

A few EA Black women discussed unique barriers to getting a prescription such as attending doctor’s appointments with their mothers. To overcome this barrier, a few EA Black women discussed preferring more on-demand PrEP options that would not require a prescription.

I keep comparing it to birth control. I feel like it’s normal only because you need a prescription to get birth control… but you don’t always necessarily need a prescription to get a Plan B. Which is like an in-the-moment thing. So maybe if there was like an in-the-moment [PrEP modality] …I know there is an in-the-moment thing because, like, it’s part of a rape kit [post-exposure prophylaxis], from my understanding. Like, they give you a pill that’s supposed to help prevent STDs and all that stuff. I feel like that could provide in the moment [PrEP], but I do think you should probably get a prescription for it. – Angelica, 22-year-old EA Black woman

### Marketing and Communication Preferences

Black women in the study discussed their PrEP marketing and communication preferences, including in-person options (e.g., forums) and virtual options (e.g., social media and commercials).

EA and OA Black women said they learn it on their own or from the doctor’s office or school nurse. EA Black women suggested bringing awareness about PrEP to sexual health education classes in schools. Both EA and OA Black women suggested social media campaigns and PrEP commercials that include Black women as potentially effective ways to raise awareness about PrEP among Black women.

…I definitely think maybe a social media campaign would help because a lot of people scroll on TikTok, Instagram, and Twitter all day long, and so if there’s a campaign or a post out there talking about it and maybe having a link to a website where they can learn more about it…. – Jessica, 18-year-old EA Black woman

In addition to social media campaigns, EA Black women recommended young women conventions, dedicated community spaces for EA Black women to discuss their sexual health, forums, and even question and answer nights on college campuses.

We have at [school] Q and A nights for dating and stuff…sex is discussed in a Q and A, but I feel like that [PrEP] should be something that’s incorporated…Like, they’re kind of destigmatizing herpes because cold sores are like a form of herpes. So, they’re kind of working towards destigmatizing that [herpes], but HIV is something that’s not even being worked on as being destigmatized. Samantha, 22-year-old EA Black woman

## Discussion

This qualitative study explored PrEP modality, service delivery, and marketing and communication preferences among emerging adult (EA) and older adult (OA) Black cisgender women in Baltimore, Maryland. We found that the biggest age-related difference between EA and OA Black women was their preferred PrEP modality, with EA women preferring oral formulations of PrEP and OA women preferring the injectable. Preferences for service delivery and marketing and communication were similar for both age groups, with both groups wanting more demand options for PrEP and increased PrEP education. EA college women, in particular, discussed the importance of having peer spaces to discuss PrEP on college campuses, whereas OA women highlighted the need for health forums in communal spaces in Baltimore. These findings can be used to develop strategies to make Black women aware of PrEP, provide them with holistic and important PrEP knowledge, and tailor PrEP uptake strategies in Baltimore.

Our study’s finding that Black women’s PrEP preferences aligned with their birth control experiences and preferences are consistent with prior research (12, 32). Oral formulations of PrEP and the injectable were the two most preferred PrEP modalities among women in the study, which is consistent with findings from Calabrese et al. (12). For example, when examining PrEP modality preferences compared to contraceptive experiences and preferences, Calabrese et al. found that women who were currently using comparable contraception modalities (e.g., oral birth control) had higher odds of preferring the same PrEP modality (i.e., oral formulations of PrEP) (12). Our study also explored preferences for the implant and ring. Though no EA Black woman preferred the ring, at least one OA Black woman preferred each modality. These findings are consistent with Irie et al. findings that women preferred oral and injectable formulations of PrEP (10). In our study, this preference is in part due to the low efficacy of the ring and uncertainties of how well it would protect them from HIV. Modalities such as the injectable may be preferred by Black women due to its non-daily nature, which would offer more discretion and help to overcome transport issues, especially for women concerned about stigma and for women who have inconsistent sexual intercourse associated with PrEP use (33). Our findings demonstrate the utility of having multiple PrEP modalities to offer Black women, as different modalities have implications to address social, financial, and structural determinants of PrEP use. For EA Black women who prefer the oral formulations of PrEP, future strategies can teach women about behavioral strategies to adhere to oral formulations of PrEP, whereas OA Black women would benefit from strategies to overcome fear surrounding injectables.

Factors concerning one’s ability to attend follow-up medical appointments affected Black women’s preferences for PrEP modalities and service delivery in this study. When asked about specifically having to return to the clinic every three months for follow-up, transport was reported as a major anticipated challenge by EA and OA Black women in the study and reduced their preference for PrEP. Transport represented a critical perceived barrier to returning for follow-up PrEP appointments every three months and diminished preference for the injectable. Many EA college women who considered PrEP questioned how they would receive care if their primary care provider was not located near the school since transport options were limited. Similarly, OA Black women discussed utilizing public transport (e.g., buses) since they did not have cars. Transport has been long established as a barrier to healthcare (34–38), especially HIV prevention and treatment (39–43). Limited or lack of transport, including inconsistent public transport, has been specifically shown to be a barrier to PrEP (43, 44). Ojikutu et al. reported that individuals had to drive more than one hour to access a PrEP provider (45). Moreover, they reported that participants who lived in areas with a higher PrEP clinic density were significantly more likely to use PrEP (45), thus demonstrating that PrEP service delivery preferences include access to PrEP prescribing facilities that would allow individuals to seek PrEP and attend follow-up care appointments. Future PrEP implementation strategies would benefit from including financial resources that could help Black women overcome these barriers to PrEP initiation and follow-up. Though EA and OA Black women expressed acceptance of needing a PrEP prescription, they expressed a preference for a more on-demand PrEP options that they can access at the pharmacy. While on demand options have been explored for men who have sex with men, they have not yet been explored for heterosexual women (46, 47). Additionally, implementation strategies such as virtual PrEP appointments and mailing PrEP to homes may help women overcome transport, as well as childcare and scheduling conflicts, that impede them from getting to the doctor’s office (48). By having PrEP available in diverse settings like the pharmacy (49), or even at community-based organizations or groups (50, 51), that are more accessible, women can make decisions around which modality works best for them and can effectively attend routine appointments for PrEP refills and follow-up testing. Targeted training with community-centered healthcare providers could also help women build rapport and trust with providers, making the sensitive conversation about PrEP easier for Black women.

EA and OA Black women discussed a need to expand PrEP marketing to not only include Black women but to also include a wider range of marketing outside of commercials and pamphlets. These suggestions have been shared by Black women in prior studies who also suggested information sharing amongst networks, community events, support groups, and the use of social media to disseminate PrEP information, which would work to normalize PrEP communications (15, 23). For example, Arnold et al. reported that Black women wanted PrEP communication through social media, text messages, and emails, with specific messaging around reproductive health or other universal women-centered language (23). This study highlighted the importance of providing holistic education for PrEP that is not solely focused on the threat of HIV (23). Furthermore, PrEP education should be inclusive of its utility for all women, including those in a relationship and married (23). Findings from a PrEP education intervention among Black college women that demonstrated an increased likelihood to consider PrEP, regardless of modality, after the intervention improved their understanding and learning about PrEP (52). Understanding Black cisgender women’s PrEP modality, service delivery, and marketing and communication preferences could help to inform tailored implementation strategies for PrEP prescribing and uptake. EA Black college women might benefit from peer groups that discuss PrEP and sexual health on campus, whereas OA Black women might benefit from community forums and clinic-based education sessions on PrEP. Moreover, EA and OA Black women could benefit from PrEP education through social media campaigns like videos and posts featuring trusted influencers in the Black community. Though limited studies focus on social media marketing specifically for Black women (53), social media marketing has been found to improve PrEP education in other populations (54). OA Black women, on the other hand, may benefit from PrEP education through secure email channels with links that connect them to blogs and online platforms to further discuss PrEP preferences and benefits, work that has not been yet explored. Age-specific PrEP implementation strategies have also been recommended by prior studies (22, 55). For example, Chandler et al. explored information that would be vital in the development of an app to provide PrEP education and communication to Black women. Though this app is still being developed, findings have important implications for how we deliver information to Black women in confidential and nonjudgmental ways. By utilizing trusted influencers in the Black community, PrEP education could be better targeted to Black women.

Our study examined PrEP modality preferences by age group for four PrEP modalities – oral formulations of PrEP, injectable, ring, and implant. It also adds to the growing body of literature discussing PrEP modality, implementation, and delivery preferences among a full sample of Black cisgender women of reproductive age. Yet, this study is not without limitations. Among our sample of EA Black women, college women comprised the majority of these women. However, college Black women were included in both the EA and OA groups. Black college women highlighted unique concerns and suggestions for PrEP modalities, service delivery, and marketing and communication based on their lived experiences attending college. However, Black women did not discuss distinctive features of injectable PrEP, such as the need to take an oral formulation of PrEP for a year after stopping injectable PrEP. We did not probe on this aspect of injectable PrEP, which may have impacted their preferences if they were given this information. Additionally, we did not give Black women statistics about the efficacy of the ring, which also could have impacted their preferences. We purposively recruited Black women through flyers, social media, and in-person events. It is also possible that our recruitment methods reached Black women who had a lower HIV risk and a greater trust in healthcare or science since they agreed to discuss HIV with a researcher. Similarly, it is possible that women in our study were more open to discussing HIV prevention strategies compared to women who did not participate in the study.

## Conclusions

Despite these limitations, these findings highlight the intersectional invisibility of Black women within the PrEP research. This is one of the few studies highlighting PrEP differences by age and its utility in tailoring PrEP implementation services to Black cisgender women in Baltimore. For example, this study highlighted an age difference in PrEP modality preferences based on contextual life factors that each age group experience. This study also highlighted college as a life experience for emerging adult Black women that may influence PrEP preferences. To improve PrEP equity and initiation among current and emerging PrEP modalities, it is crucial to better integrate the lived experiences and preferences of Black cisgender women and enhance their representation in PrEP messaging.

## Data Availability

The qualitative data generated and/or analyzed during the study are not publicly available because they contain information that could compromise participant privacy and/or consent. The corresponding author can be contacted for follow-up questions and/or concerns.

## Abbreviations

PrEP: Preexposure Prophylaxis
US: United States
LAI: Oral and Long-acting Injectable
EA: Emerging Adult
OA: Older Adult
IRB: Institutional Review Board
